# Industrial Applications, Principal Sources, and Extraction of Galactomannans: A Review

**DOI:** 10.3390/foods14091587

**Published:** 2025-04-30

**Authors:** Yaquelin Flores García, Martha Fabiola Martín del Campo Solís, Jorge H. Gómez-Angulo, Alma Hortensia Martínez Preciado, Jorge Manuel Silva-Jara, José Daniel Padilla de la Rosa, Zazil Y. Escalante-Garcia

**Affiliations:** 1 Departamento de Ingeniería Química, Centro Universitario de Ciencias Exactas e Ingeniería (CUCEI), Marcelino García Barragán # 1421, Guadalajara C.P. 44430, Jalisco, Mexico; yaquelin.flores7768@alumnos.udg.mx (Y.F.G.); jhector.gomez@academicos.udg.mx (J.H.G.-A.); alma.martinez@academicos.udg.mx (A.H.M.P.); 2 Departamento de Fundamentos del Conocimiento, Centro Universitario del Norte (CUNORTE), Carretera Federal # 23, Km. 191, Colotlan C.P. 46200, Jalisco, Mexico; martha.martindelcampo@academicos.udg.mx; 3 Departamento de Farmacobiología, Centro Universitario de Ciencias Exactas e Ingeniería (CUCEI), Marcelino García Barragán # 1421, Guadalajara C.P. 44430, Jalisco, Mexico; jorge.silva@academicos.udg.mx; 4 Unidad de Tecnología Alimentaria, Subsede Zapopan, Centro de Investigación y Asistencia en Tecnología y Diseño del Estado de Jalisco, A. C., Camino Arenero # 1227, El Bajío, Zapopan C.P. 45019, Jalisco, Mexico; jdpadilla@ciatej.mx

**Keywords:** extraction, galactomannans, polysaccharide, gum, industrial use

## Abstract

Galactomannans (GMs) are polysaccharides with diverse industrial applications due to their functional properties, such as their use in thickeners, stabilizers, and gelling agents. Their use originated in the food industry and has rapidly expanded to other industries due to their biocompatibility, biodegradability, non-toxicity, and low cost. Galactomannans can be extracted from different plant species, resulting in gums with diverse physicochemical properties. Furthermore, there are different methods for their extraction and purification, each with their own advantages and disadvantages. The structure of galactomannans determines their application in industry, so their characterization is also important. This article presents a comprehensive review of galactomannan sources, as well as their extraction, purification, and characterization methods. It also includes the main applications of these polysaccharides in various sectors.

## 1. Introduction

Galactomannans are polysaccharides found in various plant sources, mainly obtained from a wide variety of tree legume seeds. For example, these include fenugreek (*Trigonella* spp.), guar (*Cyamssopsis tetragonoloba* L.), tara (*Caesalpinia spinosa*), carob (*Ceratonia siliqua*), or mesquite seeds (*Prosopis* sp.) to name a few [1,2]. Galactomannans have the ability to absorb water and form highly viscous and stable aqueous solutions. Materials with these types of characteristics are called hydrocolloids, mucilages, or gums [3,4]. GM content varies among different seeds; for example, guar gum seeds contain up to 40–50% by weight. Fenugreek seeds typically have around 20–30%, depending on the species and growing conditions [5].

GMs are composed of galactose and mannose units, forming a linear molecular structure made up of D-mannose chains with β-1,4 linkages. Branches of D-galactose are attached via (1,6) linkages every five or four mannose units [6,7]. GMs show a mannose/galactose (M/G) ratio that varies depending on the extraction source. In their natural form, galactomannans are intertwined with other components of the cellular matrix, which prevents their functional properties from being fully utilized. Therefore, galactomannans must be extracted to release and purify them for use in various industrial applications. The extraction method also influences the M/G ratio [8].

**Figure 1:**
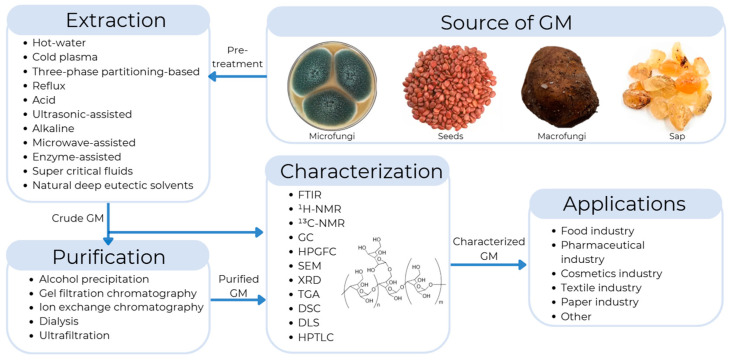
General representation of the extraction, purification, characterization, and applications of galactomannans (GMs).

Galactomannans can be extracted using various techniques, including thermal, acid, enzymatic, and microwave extraction, among others. Each technique offers different levels of efficiency as well as advantages and disadvantages in terms of purity and operating costs. The choice of extraction method will largely depend on the final application of the galactomannan. For example, these polysaccharides can be applied in various sectors, where they are used as stabilizers in food. They are used as excipients in the pharmaceutical industry, thickeners in cosmetic products, biopolymers in biodegradable packaging, and reinforcing agents in paper and textile manufacturing (Figure 1) [9].

This article presents a comprehensive review of the sources of galactomannans. It presents extraction methods such as hot water, ultrasound, microwaves, chemical, and enzymatic methods. It also discusses purification methods such as precipitation, dialysis, ion-exchange chromatography, and ultrafiltration. Various techniques for the characterization of galactomannans are presented, such as Fourier transform infrared spectroscopy (FTIR), nuclear magnetic resonance (^1^H NMR and ^1^C NMR), and high-performance gel filtration chromatography (HPGFC).

## 2. Galactomannans

Galactomannans are multifunctional polysaccharides found mainly in the endosperm of seeds of various plants such as tara (*Caesalpinia spinosa*), fenugreek (*Trigonella* spp.), carob (*Ceratonia siliqua*), guar (*Cyamopsis tetragonoloba* L.), or mesquite (*Prosopis* sp.) (Table 1) [1,2].

Galactomannans are synthesized in plants from the products of photosynthesis. During photosynthesis, the Calvin cycle generates glucose. Mannose is synthesized from glucose-6-phosphate via the hexose interconversion pathway, which is converted into GDP-mannose, a fundamental precursor in the formation of β-mannan [80]. Glucose is also transformed into UDP-galactose, which is used by galactosyltransferase to add galactose to the β-mannan chain. The amount of galactose incorporated gives rise to the galactomannan structure and varies depending on the plant species. Galactomannans are stored in the cell wall or in the endosperm of seeds, where they serve as an energy reserve for the plant.

**Figure 2:**
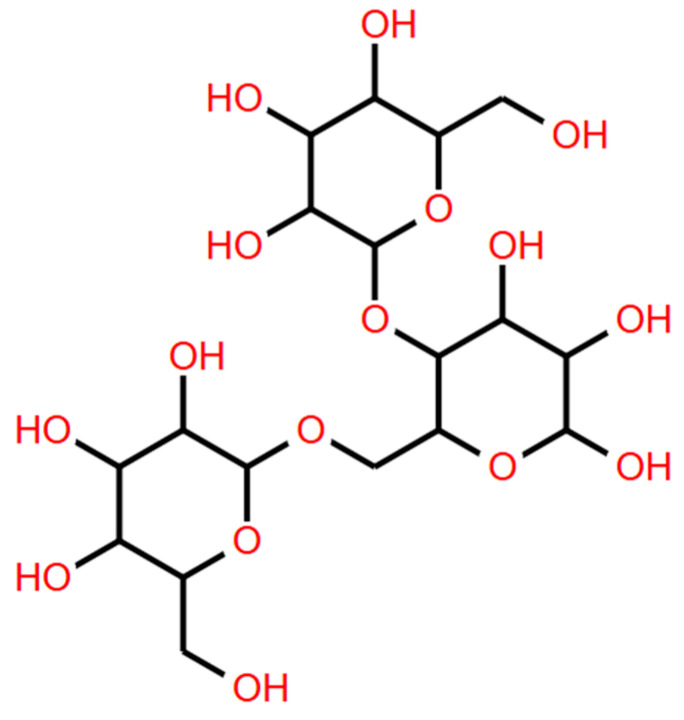
Schematic representation of the chemical structure of galactomannans. (Structure created with the Biomodel program).

Galactomannans are of great industrial importance [8]. The structural characteristics of galactomannans give them properties such as high solubility in water over a wide range of temperatures. The plant cell wall is composed of various polysaccharides, such as cellulose, hemicelluloses, and pectin. Among these, cellulose is the most abundant, constituting 30 to 50% of the total dry mass of the cell wall. Hemicellulose constitutes 20 to 35% of it [81]. Hemicelluloses are grouped into xylans, xyloglucans, and mannans. Galactomannans are a subclass of mannans and are multifunctional macromolecular carbohydrates. Galactomannans are made up of the D-mannopyranose main chain linked by β (1→4) glycosidic bonds with D-galactopyranose branches linked to the mannan main chain by α (1→6) bonds (Figure 2) [9].

**Figure 3:**
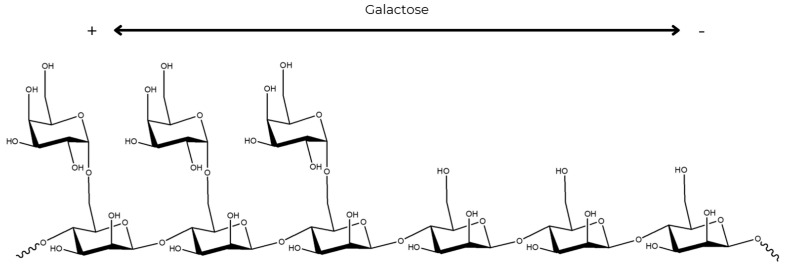
Representation of the substitution of galactose in the mannose chain. (Structure created with the Chemdraw program).

The galactose units are organized as random doublets and triplets along the main chain [5,82]. Such random substitution of the galactose units results in regions of high and low substitution in the mannan main chain (Figure 3). In galactose-sparse regions, non-covalent interactions occur between polymer chains. In galactose-abundant areas, high substitution hinders the formation of more organized structures. This is due to steric hindrance of the side chains, which also leads to high solubility in aqueous solutions [83].

Galactomannans are distinguished from each other by the M/G ratio, which varies from approximately 1.1 to 3.5. The M/G ratio is directly related to the structure and function of galactomannans.

Furthermore, galactomannans are susceptible to molecular changes due to the number of hydroxyl groups (-OH) and the absence of ionic charges in their structure [84]. Studies indicate that there is a correlation between the bioactivity of these polysaccharides and their structural characteristics. These characteristics include the degree of substitution, the position of the substituent, the molecular weight, and the conformation of the chain [85].

### Types of Galactomannans

The basic structure of galactomannans is a mannose backbone with galactose branches. However, significant structural variations may occur depending on the source. These variations include the ratio of galactose to mannose, the distribution of galactose branches, and the molecular weight. These differences directly influence the functional properties of galactomannans, as well as their application. These structural changes allow galactomannans to act as gum, hydrocolloid, or mucilage [86].

**Figure 4:**
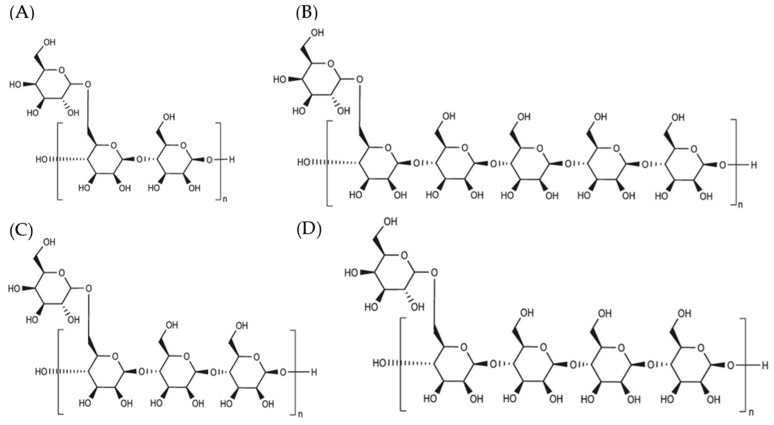
General chemical structures and repeating units of galactomannans: (**A**) guar gum, (**B**) cassia gum, (**C**) tara gum, and (**D**) algarroba gum [89].

The general term to describe the behavior of galactomannans is gum and GMs can be soluble or insoluble in water. This term is also used to refer to polysaccharides of plant origin such as locust bean gum and guar gum. On the other hand, mucilages do not dissolve easily in water and form viscous masses, unlike hydrocolloids, which dissolve easily, forming solutions [87]. Most hydrocolloids offer high viscosity with low concentrations, around 1%, and are capable of forming gels [88]. Galactomannans stand out among other gums because they offer great viscosity retention and great emulsifying and stabilizing capacity. Therefore, they are primarily used to alter rheological behavior [82]. The structural characteristics of galactomannans, mainly hydroxyl groups and hydrogen bonds, provide specific rheological and physicochemical properties, determining their functionality (Figure 4) [10,82].

Water solubility is the basic characteristic affected by the structure of galactomannans. For example, the mannan main chain is insoluble while the galactose side chains are the ones that impart solubility to the molecule. In galactomannans with fewer galactose chains, the mannose units of the main chain come closer together to form intrachain hydrogen bonds, which leads to a decrease in water solubility [86]. Consequently, the solubility of galactomannans will be proportional to the reducing galactose side chains per repeat unit. In turn, this ratio will depend on the source from which the galactomannans are obtained, for example: ~30% for locust bean gum (M/G ratio: 4/1), ~60–70% for guar gum (M/G ratio: 2/1), and ~80% for fenugreek gum (M/G ratio: 1/1) [2,17,31,90,91,92].

These structural changes also affect the basic physical properties of galactomannans: the fiber content is 1% for locust bean gum, followed by tara gum with 2%, guar gum with 2–3%, and fenugreek gum with up to 4–6%. Likewise, the protein content is affected, decreasing as the mannose decreases: for locust bean gum, it is 4.57%, guar gum 3.46%, and fenugreek gum 2.62% [86]. The distribution of galactose branches along the mannose chain also varies according to the source of the galactomannan. For example, uniform branches such as guar gum give rise to a more stable and reproducible viscosity, while galactomannans with an irregular galactose distribution such as locust bean gum offer greater synergy with other gelling agents [85].

After the galactomannans dissolve, the viscosity-producing effect, which is affected by factors such as thermal processing, ionic strength, pH, and the neutral character of the galactomannans, begins to occur [93]. Galactomannans with a lower number of galactose side chains offer lower viscosities, although the thickening capacity also depends on the molecular weight and chain length of the polymers present in the molecule. Due to their different molecular weights, fenugreek gum has the highest thickening capacity, followed by guar gum, tara gum, and locust bean gum [8,10,90,94]. Galactomannans that have fewer galactose chains in their structure have greater gelling properties due to their unsubstituted mannose blocks, which chemically interact with polysaccharides. The gelling capacity in terms of lowest to highest strength is fenugreek gum, guar gum, tara gum, and locust bean gum. Galactomannans with a higher proportion of mannose form stronger gels when combined with other hydrocolloids, while guar gum forms more viscous solutions but with less gelling capacity [10,90,95].

The thickening capacity is related to the applications of galactomannans and will define the required concentration. For example, at low concentrations, the polymers move freely, exhibiting Newtonian behavior with constant viscosity independent of the shear rate. At a critical concentration, the polymer movement is restricted, resulting in non-Newtonian behavior [96].

Galactomannans are a type of polysaccharide that may play a role in the structure of cell walls. They can be broken down into monosaccharides such as galactose and mannose, which can then be used as an energy source by plant cells. Galactomannans have different beneficial activities and can be applied in various industries, so their extraction becomes relevant.

## 3. Extraction Methods

Existing extraction methods have disadvantages in the process, and in addition, the proteins present in the matrix have an impact on the purity of the galactomannans [97]. Therefore, it is important to eliminate them from the beginning of the process. They can be removed by selective precipitation with 30–50% ethanol or isopropanol. Membranes can also be used to separate proteins (<10 kDa) or enzymes that remove proteins without affecting polysaccharides such as bromelain, pepsin, or trypsin can be used. Adjusting the pH to 4–5 before extraction can also be beneficial [98].

An appropriate extraction method is a critical factor that affects the bioactivity of galactomannans, being a key to having a high yield and maintaining physicochemical characteristics such as functional properties, molecular weight, protein, and galactomannan content [99,100,101,102]. There are various methods for the extraction (Table 2) and purification of galactomannans, and each one produces different structures, which are reflected in a different biological activity [103]. Alternative methods should be used in circumstances more suitable for existing processing techniques [104,105,106].

The main objective of choosing an extraction method should be focused on a fast process that contributes to the economy and the ecosystem.

### 3.1. Hot Water Extraction

The polysaccharides extracted by this traditional method are mainly neutral polysaccharides or polysaccharides that remove insoluble substances directly or by centrifugation [118,119,120]. The extraction rate depends on the source of galactomannans under different extraction conditions such as extraction temperature, extraction time, and solid–liquid ratios. Each source has different optimal extraction conditions [121]. Extraction temperature and time have a positive effect on extraction yields; high temperature or long extraction time may destroy the structure of polysaccharides. On the other hand, a higher liquid–solid ratio increases the mass transfer from solid to solvent and improves solvent diffusivity in cells. However, increasing the solution volume under a higher liquid–solid ratio also affects the purification process and production costs [122]. You can start with an intermediate ratio (15:1–20:1 mL/g) and adjust according to yield. Applying ultrasound or maintaining constant agitation during extraction can improve solubilization without increasing the amount of water, which would reduce costs. Balancing these factors requires systematic optimization using experimental design techniques, such as response surface methodology (RSM). By analyzing variables such as temperature, time, and cartridge ratio in an integrated manner, it is possible to determine the most efficient extraction conditions for each polysaccharide source.

This method has advantages such as low cost, easy operation, and the lack of requirement for sophisticated equipment, and it can be performed on an industrial scale. However, it is a method with low efficiency and requires a lot of time. This method is usually accompanied by other extraction methods to optimize the extraction, and different factors are considered for the selection of various substances [123].

### 3.2. Cold Plasma Extraction

This method is also known as non-thermal plasma and is an environmentally friendly, cost-effective, and energy-efficient technique [124]. This method is based on ionization, stimulation, and separation of gases [125]. Basically, it contains electrons with an excessively higher temperature than that of heavy particles (ions and neutrals) [126]. This is because the cooling of ions and uncharged molecules is more effective than the energy transfer of electrons [127]. The temperature of the cold plasma gas does not increase and remains in a state of thermodynamic disequilibrium [128]. Therefore, cold plasma is ideal for application in biological material modifications in terms of surface rupture, wettability, and roughness [129,130]. These are characteristics mainly related to the migration of the substances they contain from the interior to the surface during extraction [131,132]. In this way, according to the plasma parameters (type of gas, input energy, pressure, power) and the inherent characteristics of the substrate, different chemical and physical modifications of the surface are generated [133].

Cold plasma treatment can cause ruptures on the surface of the seeds and decrease the pH of the extraction solution, resulting in an increase in the extraction yield of approximately 67–122%. Galactomannans extracted by this method show higher water binding capacity, higher apparent viscosity, and higher swelling index due to structural disintegration caused by the cold plasma treatment [30].

### 3.3. Extraction Based on Three-Phase Partitioning

Three-phase partitioning is applied to the extraction and separation of compounds such as proteins, lipids, carbohydrates, and small-molecule organic compounds. This method involves precipitation interactions with salts, cosolvents, isoionic material, etc. [110]. This technique consists of an upper phase (t-butanol) containing lipids, pigments, and hydrophobic materials, a lower aqueous phase containing polysaccharides and polar components, and an intermediate phase enriched with proteins [109]. The main factors influencing this extraction are the mass fraction (ammonium sulfate), the mass fraction of t-butanol, temperature, and pH. The “salting-out” effect is the force that drives the partitioning of polysaccharides with a low mass fraction of ammonium sulfate. The optimal pH value in this extraction is related to the isoelectric point of the sample. This process is usually carried out at room temperature, but it is of vital importance. For example, an increase to 20 or 40 °C provides an improvement in the mass transfer rate and facilitates hydrogen bond formation. This results in an improvement in the hydrophilicity of the extracted polysaccharides, making them more concentrated in the lower phase. To improve TPP extraction, salt precipitation efficiency can be increased by using an ammonium sulfate concentration of 20% to 40% (*w*/*w*). A pH of ~5–6 can improve galactomannan recovery. Additionally, ultrasound (20–40 kHz) can be applied for 5–10 min to increase mass transfer and reduce extraction time. The alcohols in the upper phase can also be replaced with isopropanol or ethanol [121].

### 3.4. Thermal Reflux Extraction

This method is mainly used for the extraction of polysaccharides and promotes dissolution, solvent penetration, and diffusion of the polysaccharide through thermal effects [102]. The performance of this method commonly depends on the extraction time and the temperature used [134]. It is a method that requires a relatively long extraction reaction time, approximately at least 2 h, and the yield and purity of the product are not ideal [135]. It is a simple and inefficient method that causes the degradation of the polysaccharides and considerably decreases their pharmacological activity if the temperature is not controlled [136,137]. Reflux extraction is an effective extraction method to obtain good yield and bioactivity, with higher polyphenol content and greater weight, presenting better antioxidant properties as long as low temperatures are used [138]. Reflux extraction has the advantage of using little solvent, since, as its name suggests, the solvent itself evaporates and condenses without the need to add more. There are different ways to apply this method: the traditional one (Soxhlet) or combined with other techniques such as MAE. In this way, microwave energy is applied to heat the sample and the solvent, combining these techniques to reduce extraction time. It is also possible to combine this technique with UAE or deep eutectic solvents (DESs) to avoid compound degradation or make it a sustainable method, respectively.

### 3.5. Alkaline or Acid Extraction

This method is suitable for hard seeds because it is capable of degrading the structure of the thick fibers, destroying the cell walls. It also hydrolyzes the ester bonds between the cell wall protein and the glucan, which further increases the release of polysaccharides [139]. Alkalinity or acidity must be strictly controlled throughout the extraction because glycoside bonds in polysaccharides may be broken, and some polysaccharides are hydrolyzed when alkalinity or acidity is high. Once the extracts are obtained, they must be neutralized or dialyzed, concentrated, and precipitated immediately [140].

The alkaline extraction method increases solubility by forming salts with acidic polysaccharides. Therefore, this method is suitable for the extraction of uronic acid-containing polysaccharides and acidic polysaccharides. The alkali-soluble fractions consist especially of highly branched β-(1→3, 1→6)-glucan [141,142]. The most important factors influencing the extraction rate are alkali concentration, temperature, and extraction time [121].

### 3.6. Ultrasound Extraction

Ultrasound is one of the most effective techniques for polysaccharide extraction based on applying ultrasonic waves in the frequency range of 20–100 kHz [143]. Ultrasound produces and transfers a large amount of energy, causing the medium to accelerate to a state of high-speed vibration, which produces a cavitation effect in the liquid. In other words, under the action of considerable destructive stress, a cavitation bubble forms; this bubble swells and bursts instantly. The absorbed sound field energy is released in an extremely short time and in an extremely small space, generating high temperature and high pressure at the same time [97]. Strong shock waves, together with micro-sound waves, end up rapidly breaking the cell wall structure. The effective components of plant cells are released into the solvent, where they mix completely, accelerating diffusion and thus improving the extraction rate [144]. This technique produces a coagulation effect, thermal effect, biological effect, chemical effect, emulsification, grinding, and diffusion as side effects. This can result in a product with low solubility, purity, functionality, and viscosity. Therefore, maintaining a controlled temperature, power, and flow rate is essential to minimize these effects. Furthermore, it is recommended to use short pulses instead of continuous ultrasound exposure [143].

This method considerably reduces extraction times, has a high extraction rate, and reduces interference from other solvents. Despite this, it is a technique that is not suited to large-scale application due to the high cost of the required equipment [144].

### 3.7. Microwave Extraction

The extraction principle of this technique is based on the fact that micro-rays irradiate the solvent and move into the cell through the cell wall. The frequency of the microwaves is between 300 MHz and 300 GHz. As the solvent and the cell absorb the micro-rays, the temperature and pressure increase. The cell wall breaks when the pressure exceeds its capacity, releasing and transferring the components to the solvent. This method offers a high extraction rate, short extraction time, and high heating efficiency and is able to protect the active components from destruction [145]. It can also be combined with other techniques such as enzymatic hydrolysis or hot water extraction.

### 3.8. Enzymatic Extraction

This method has been widely used in recent years because enzymes help to reduce the extraction condition, are able to degrade plant tissues under subtle conditions, and accelerate the release and extraction of polysaccharides [146]. This technique is very useful because it also breaks down irrelevant compounds such as pectin, protein, and starch, among others. The main condition to consider for an efficient process is to selectively hydrolyze the glycopeptide bonds in the glycoproteins, controlling this aspect in order to not significantly degrade the polysaccharides by using specific enzymes. These are capable of hydrolyzing structurally complex polysaccharides into simple fragments. The most commonly used enzymes are hydrolases that include protease, cellulase, pectinase, papain, α-amylase, β-1,4-xylanase, and β-1,4-mannanase. The most important enzymes for the hydrolysis of mannan includes endo-β-mannanase (EC 3.2.1.78), protease (EC 3.4.21.62), endo-1,4-β-glucanase (EC 3.2.1.4), endo-1,4-β-xylanase (EC 3.2.1.8), exo-β-mannosidase (EC 3.2.1.25), pectinase (EC 3.2.1.15) β-glucosidase (EC 3.2.1.21), cellobiohydrolase (EC 3.2.1.91), acetyl mannan esterases (EC 3.1.1.6), and α-galactosidase (EC 3.2.1.22), and 1,4-β-xylosidase (EC 3.2.1.37) [147]. It is also possible to combine several enzymes to obtain a better performance, taking into account that the type of enzyme, the enzyme concentration, and the pH are highly relevant factors that influence the extraction efficiency. For example, the combination of β-mannanase and α-galactosidase is very efficient in breaking down galactomannans into simple components. The combination of cellulase and pectinase enhances the release of galactomannans from plant matrices [121]. Microorganisms are the sources of the biological production of enzymes that are obtained through microbial fermentation (Table 3). Fermentation offers advantages such as low production costs, high efficiency, and a short production cycle [33].

In general, it is an efficient technique because it offers high specificity and broad enzymatic catalytic activity, making it an optimal extraction option [154].

### 3.9. Extraction with Supercritical Fluids

This extraction technique also has a high extraction rate and provides high product purity, which leads to faster separation, purification, and reduction of the production cycle [155]. The use of this extraction method requires less use of solvents and fast removal times, and it also involves regulating the dissolution force with the help of adjusting its mass since the physicochemical characteristics are between gas and liquid [156]. Taking care of this relationship is vital because even a slight adjustment in the pressure and/or temperature parameters will significantly modify the density of the fluid and increase the dissolution force by approximately 80 to 100 times [157,158]. This is because the molecular propagation rate of supercritical fluids is as high as that of gas and the solubility density is as strong as that of liquids [105]. Overall, it is a non-toxic extraction method and offers higher selectivity and extraction yield by tailoring the operational criteria. More specifically, the selectivity and purity of the final extract can be modulated by varying the temperature, pressure, and flow rate of supercritical fluids [116,159].

### 3.10. Natural Deep Eutectic Solvent Extraction

Natural deep eutectic solvents are based on hydrogen bond acceptors (HBAs) and hydrogen bond donors (HBDs). These solvents have unique properties of chemical and thermal stability, low vapor pressure, low melting point, non-toxicity, and low costs [160].

NADES are suitable for the extraction of polysaccharides due to their ability to donate or accept external protons or electrons [161]. NADES play a relevant role in the extraction of polysaccharides; water helps to reduce viscosity and facilitate mass transfer, thus improving extraction efficiency. However, excessive water content can destroy hydrogen bonds and decrease the extraction efficiency of the method. This technique shows a higher yield than the thermal method [121].

## 4. Optimization of the Extraction Method

The application of a single method for the extraction of polysaccharides is limited; a composite method can significantly improve the extraction rate and reduce the cost [97]. For example, studies have shown that ultrasound-assisted enzymatic extraction provides better polysaccharide yields and shortens the extraction time (from 4 h to 32 min). That is, by combining the techniques, higher yields are observed in less time than with the enzymatic technique alone. The average molecular weight of the polysaccharides extracted with ultrasound (343–473 kDa) was lower than the polysaccharide extracted without ultrasound (500–620 kDa), suggesting that some compounds underwent depolymerization [162].

Another technique used in the food industry for the separation of bioactive ingredients is ultrasound-assisted triphasic partitioning. TPP is an environmentally friendly, rapid, and efficient extraction method [163]. The principle of TPP involves mixing crude extracts or suspensions with solid salt and organic solvent to form three distinct phases simultaneously. This method has been used to extract and purify enzymes, lipids, and proteins [164]. The ultrasound-assisted triphasic partitioning process has also been studied and is highly effective in the extraction of polysaccharides. The yield obtained was higher (112%) than with the ultrasound and three-phase partition methods separately with 60 and 93%, respectively.

Furthermore, the extraction time is shorter, at only 10 min. Ultrasonic extraction requires 60 min, while three-phase partition extraction requires 30 min [165]. The ultrasound method can be performed using NADES to improve the extraction yields of polysaccharides. The most important factors are ultrasonic power, temperature, extraction time, and the solvent–solid ratio. In an extraction time of 40 min, high yields were achieved, higher than those achieved with hot water extraction under the same conditions [166].

Techniques such as ultrasound have also been combined in extraction processes with pressurized liquids; although this approach has been little studied, it is a complex process in which different mass and energy transfer mechanisms interact with each other [167]. Understanding the individual and combined impact of each of the process variable extraction processes remains a major challenge for researchers.

To achieve an optimal balance between extraction speed, cost-effectiveness, and environmental sustainability, it is essential to develop combined methods that improve process efficiency while maintaining the integrity of the extracted compounds. Approaches such as enzyme-assisted extraction, environmentally friendly solvents, and emerging technologies such as supercritical fluid extraction or microwave-assisted extraction could help reduce energy consumption and solvent usage while improving galactomannan purity. Furthermore, strategies to minimize protein interference, such as selective precipitation, membrane filtration, or enzymatic hydrolysis, should be integrated into the extraction process to improve purity without compromising yield.

## 5. Galactomannan Purification Techniques

After the extraction of galactomannans, crude galactomannans are obtained, which may contain a large number of impurities. These impurities generally come from other components present in the plant matrix from which the galactomannans are obtained. Due to the incomplete separation of the germ layer and endosperm, unwanted compounds are partially extracted during the process.

Also, depending on the solvent used for extraction, residues may be present in crude galactomannans. To avoid this contamination, careful manual separation is recommended. The most common impurities are proteins, lignins, salts, minerals, ash, and lipids [168]. These compounds are present in varying amounts depending on the extraction source. For example, protein amounts of 3.74% to 13.9% have been reported for crude fenugreek gum [169,170]. The presence of these compounds can interfere with the functional properties and quality of galactomannans, so a purification process is essential to obtain a high-quality final product [170]. Purification methods must be effective in removing these impurities without damaging the galactomannans or compromising their functional properties.

Among the main methods for the purification of galactomannans are ethanol/isopropanol precipitation, ion exchange chromatography, gel filtration chromatography, dialysis, and ultrafiltration (Table 4). They should be selected based on the nature of the impurities present and the characteristics of the extracted galactomannans. For example, dialysis or precipitation are generally used to remove proteins and salts. For simple sugars, ion exchange chromatography or gel filtration chromatography are used. For lignin, ash, and minerals, ultrafiltration can be used [168].

To obtain a high efficiency in the purification of galactomannans, factors such as sample concentration, flow rate, solvent purity, and solvent selection must be considered [177]. Generally, techniques are combined to remove impurities, proteins, and other components, although this process can be adjusted depending on the final application of the galactomannan. For example, unpurified galactomannans are commonly used in the pharmaceutical and cosmetic industries [178]. This is due to the fact that during purification, the formation of hydrogen bonds in the galactomannan structure increases, resulting in changes with amorphous transitions, which influence the mobility and functional properties of the gum [179]. Studies have reported that crude galactomannans show higher values of permittivity, conductivity, and losses compared to purified galactomannans [59].

The structure of galactomannans is complex and can be very diverse due to the many variables such as the source of extraction, the extraction method, and the purification method [180]. The characterization of galactomannans is important for their application, because their biological function is directly related to the structural characteristics of the gum [181].

## 6. Galactomannan Characterization Techniques

Understanding the structure of galactomannans can optimize their applications in industry and improve extraction and purification processes. Furthermore, by understanding their structure, we can understand how they interact with other compounds and design materials with specific properties [117]. For example, polysaccharides with a higher molecular weight have a greater hydrodynamic volume, higher viscosity, and a more complex structure [182]. On the other hand, low molecular weight polysaccharides are more likely to dissolve in water and are easier to absorb [183,184].

There are different techniques for the analysis and characterization of the structure of galactomannans, such as Fourier transform infrared spectroscopy (FT-IR), nuclear magnetic resonance (NMR), high-performance liquid chromatography (HPLC), and gas chromatography–mass spectrometry (GC-MS) [185]. Table 5 summarizes the main techniques for the structural characterization of galactomannans.

## 7. Applications of Galactomannan

Among the functional properties of galactomannans, their water retention capacity, non-toxicity, non-ionicity, high molecular weight, and solubility stand out, which allows them to be widely used in industry. Galactomannans have different applications, for example, as binders, excipients, fat substitutes, thickeners, gelling agents, plasticizers, emulsifiers, stabilizers, edible coating, and flavor encapsulants, and for the alteration of ice crystallization and the adjustment of freezing and evaporation rates [8,36,90]. All its previous qualities widely allow its use in various industries such as food, cosmetics, textile, paper, and pharmaceutical [41].

### 7.1. Food Industry

Galactomannans obtained from sources such as tara gum are included in the European Codex Alimentarius Commission (Codex) system. Therefore, their use as a food additive is approved. This includes their use in controlling flavor release and inhibiting sugar crystal formation in various products [189]. In general, galactomannans improve the texture of foods [190].

They are used in dairy products such as cream and yogurt to increase viscosity and texture, resulting in a higher quality product. The concentration of galactomannans is proportional to the creaminess of yogurt; adding gum at 0.41–0.43% increases sensory characteristics and also increases its stability and prevents sediment and whey separation [191].

It is widely used in beverage manufacturing because galactomannans are soluble in cold temperatures and are stable at low pH. Gum is also used as a source of soluble fiber, reducing calories. Adding gum at 0.1% prevents pulp in juices from precipitating, controls viscosity, thickens, and increases shelf life [189].

Galactomannans applied in breadmaking can improve chewiness. Gluten proteins (gliadin and glutenin) interact with galactomannans, creating a more flexible network and improving the bread’s texture. The bread also retains more water, making the crumb moister and softer, which creates a more pleasant chewing sensation [192]. Adding galactomannans to bread extends shelf life because it forms a gel that prevents the formation of crystals that occur during the gluten retrogradation process, leaving the bread with a fresh, soft texture for longer [193,194]. Furthermore, galactomannans have been reported to form a film on the surface of the bread, making it difficult for pathogenic microorganisms to penetrate. Overall, galactomannans improve the acceptability of bread by increasing its softness, dough elasticity, crust characteristics, chewiness, and shelf life, while reducing hardness [11].

Galactomannans present bacteriostatic and antioxidant activity that help preserve food by inhibiting the growth of microorganisms, thus extending the shelf life of various products, showing potential as a natural preservative. Galactomannans have also been applied to sausage manufacturing, improving the products’ texture, sensory characteristics, and storage time [195]. In ice cream, where a concentration of 0.3% is recommended, GMs are also used as a stabilizer due to their water-binding properties, improving texture and homogeneity [196]. Galactomannans are also used in sauces, dressings, and mayonnaise to prevent compounds from settling, improve texture, reduce syneresis, and increase viscosity [197].

In addition, galactomannans have the ability to be used as edible films or coatings such as biodegradable packaging. Polysaccharides applied as coatings contribute to reducing water loss and microbial growth, and they completely degrade non-toxic substances [41,198,199]. These coatings are mainly applied to fresh vegetables and fruits, and it has been shown that they could reduce moisture evaporation from them, improving their quality and extending their shelf life. This is due to the fact that they act as an oxygen barrier, preventing colors, lipid ingredients, and flavors from oxidizing [117]. Galactomannans represent an environmentally friendly alternative by reducing the use of conventional plastics and minimizing their environmental impact.

### 7.2. Pharmaceutical Industry

In the pharmaceutical industry, the use of galactomannans for medicines stands out due to their important properties such as being chemically inert, non-toxic, cheap, non-immunogenic, and odorless and having better solubility and stability [200]. In addition, polysaccharides are biodegradable and biocompatible. It is difficult for them to penetrate into the blood, which indicates that the appropriate dose of the drug can be administered to the precise organs and tissues at the right time [201].

Polysaccharides are used as a matrix in the formulation of tablets to protect active substances from stomach conditions and small intestine acids, allowing their release into the colon under alkaline conditions [114]. The structure of galactomannans has various functional groups in its structure, which can be converted or modified into hydrogels, allowing different drug delivery systems to be obtained [202]. The interaction of enantiomers with the excipient can lead to differentiated drug delivery rates for each enantiomer of the drug. Hydrogels can then be exploited for slow release and increased drug bioavailability [114]. It has also been shown that they can be used as antibacterial medical dressings that promote wound healing [70].

The controlled release of diclofenac sodium was evaluated in capsules and tablets using galactomannans. The drug release performance was compared with a commercial controlled-release product. The tablets resulted in a zero-order drug release; gel erosion controlled the release rather than the diffusion. On the other hand, the capsules were of a first-order model. The results obtained demonstrate the potential of galactomannans as release-retarding materials. The polymer concentration results in a decrease in drug release, and all formulations with gums demonstrated an excessive sustained release effect [203,204]. Galactomannans were applied in fast-dispersing ibuprofen tablet formulations, which are mainly used in elderly patients and in children who have problems swallowing conventional capsules or tablets. This new technology provides a high drug loading, has an acceptable taste, and leaves a minimal residue in the mouth after administration [205].

A study was conducted comparing the micrometric properties of paracetamol granules using different binders (8% *w*/*w*). The tablets in which galactomannans were used showed superior flow properties. They also showed a high resistance to crushing of the granules compared to other binders, which translates into a greater uniformity of the granule size. The use of galactomannans potentially stands out as a pharmaceutical binder [204]. There are companies that develop drugs using the different gums that have been approved. Without a doubt, galactomannans offer a wide potential for use in the pharmaceutical area because their versatility allows their application in novel developments for various drug delivery systems. However, the variability of the molecular weight and the degree of purity of galactomannans derived from the different sources and extraction methods is a problem to be solved.

### 7.3. Cosmetics Industry

There are sixteen galactomannans obtained from different sources that are approved in the international cosmetic ingredient manual. The main function of galactomannans is as hair and skin conditioning agents, and they are also used to increase the viscosity of cosmetic products. The ingredients included are gums extracted from *Cyamopsis tetragonoloba*, *Ceratonia siliqua*, *Caesalpinia spinosa*, *Trigonella foenum-graecum*, and *Cassia tora*, among others [206,207].

In this industry, galactomannans fulfill the function of emulsion stabilizers, fragrances, binders, film formers, antistatic agents, adhesives, and emollients. The gums have demonstrated excellent compatibility with almost all the main ingredients used in cosmetics, such as sodium chloride and magnesium sulfate [89]. In tests, it was observed that after 48 h, the viscosity increased slightly, while maintaining the appearance. Galactomannans obtained from *Caesalpinia spinosa* show stability in a pH range of 3 to 12, without altering the appearance or viscosity. In addition, ethanol can be added at a maximum of 10% to the galactomannans even if the viscosity increases significantly [208].

*Caesalpinia spinosa* gum demonstrated excellent compatibility in general with natural and synthetic rheology modifiers. In addition, a synergistic effect was observed with xanthan gum, forming a compact gel. In general, *Caesalpinia spinosa* galactomannans improved the sensory qualities of the gels, providing them with greater smoothness, less stickiness, velvety feel, extension, and thickness [89]. As for surfactants, it offers an improvement in the quality of the foam, without the risk of toxicity, and a thicker and softer sensation during the massage. These qualities make the gum the ideal ingredient in formulations of delicate cleaning products such as baby hygiene products and products for hypersensitive skin.

Also, the gum shows great compatibility with the conditioner cetyltrimethylammonium chloride, making it suitable for the formulation of hair products, such as cleansers and conditioning masks [209]. The gum can also be applied in makeup formulations because it shows ideal compatibility with various pigments, such as iron oxides, titanium dioxide, and zinc oxide. Even inorganic UV filters such as ZnO and silica-coated TiO_2_ show compatibility with this gum [210]. Hydrotopes such as glycerin, betaine, propylene glycol, 1,3-propanediol, and isopentyldiol show excellent compatibility and higher viscosity is observed in aqueous dispersions. When the gum is moistened with hydrotopes, the water is easily dispersed, facilitating the swelling process [208]. Galactomannans have been used in concentrations of up to 93% in hair straightening products. It is used in powders and sprays at a concentration of up to 0.05%. Likewise, another advantage of using gums is that those products that contain them are safe to apply even several times a day and can come into contact with the skin or hair for long periods after application. For example, hair sprays that contain gums, being in aerosol form, could present a risk of inhalation. Aerosols release particles below 10 mm because traces would be deposited in the respiratory tract, in the nasopharyngeal and bronchial area; therefore, gums would not be respirable; they could not enter the lungs [211].

### 7.4. Textile Industry

Galactomannans are used in the textile industry for their thickening properties. Galactomannans were initially used primarily in printing pastes, but their other properties such as biocompatibility and biodegradability have been exploited in different textile applications [212].

Printing is an essential technique used for the coloring of textiles. In this technique, a viscous paste is used where thickeners are essential to adhere the dyes to the indicated places according to the pattern [213]. The thickeners used must have a high molecular weight, stability, colorless structure, high viscosity, long hydration duration, and good storage capacity. There are thickeners of synthetic or natural origin. Synthetic thickeners are generally used in pigment printing, but they have different harmful effects on the environment. For example, bottles cannot be dumped in sanitary landfills, produce harmful gases, and cause air pollution [212]. On the other hand, natural thickeners gain importance because they are environmentally friendly, thus minimizing these side effects [214]. For this reason, galactomannans have gained great relevance within the textile industry. Guar gum is the most widely used thickener in printing pastes because it disperses in cold water, representing a great advantage. Guar gum is generally used at concentrations of 0.5% to 2% in printing pastes. For textile finishes, it is used at concentrations of 0.1% to 1% [215]. The viscosity of guar gum decreases when shear rates increase, as in most high molecular weight polymers, making it ideal for carpet printing [216,217].

Guar gum is also used for silk and wool printing, showing great fixing capacity, penetration, and color fastness, comparable with commercial thickeners such as alginate [218,219]. There are studies aimed at modifying the characteristics of galactomannans aimed at increasing the solubility and improving the swelling rate of gums [217]. The suitability of carboxymethyl guar derivatives has also been studied. It has been shown that the color intensity in printed samples varies according to the reactive dye used, the nature of the thickener, and the printing time and fixation. Depending on the degree of substitution (DS), the printed samples exhibit different characteristics such as rough touch (DS: 0.77), soft touch (DS: 1.27), or higher color efficiency [86]. All of the above makes guar gum an ideal thickener for textile printing; it is also economical and does not pose any ecological danger [220]. Locust bean gum, also used as a thickener in the textile industry, is a non-ionic polysaccharide and has a pH of 3 to 11, so it can be used in alkali-resistant printing pastes and is suitable for use with all types of printing pigments. Locust bean gum is effectively removed in post-printing washes, resulting in soft printed textiles [221]. In a study, Indalca gum (modified locust bean gum) and Arabic gum were compared by testing different printing parameters such as colors, touch, fastness, resistance, and costs. Indalca gum showed advantages for silk printing, including higher color resistance compared to gum Arabic gum-treated fabrics [222].

Fenugreek gum and tara gum were also investigated as thickeners. Hebeish et al. (2010) studied tara gum for printing cotton fabrics, resulting in higher color efficiency using tara carbamate than those obtained using conventional thickeners [223]. In another study, it was possible to isolate galactomannan gum and natural dye from tara seeds simultaneously, demonstrating the usefulness of this paste for printing cotton, wool, and silk fabrics [224]. Other sources of galactomannan, such as Cassia obovata seeds, have also been studied and are reported to be an ideal thickening agent for printing on polyester [225]. In parallel, galactomannans from sesbania seeds have proven to be very useful as thickeners [225,226,227].

Textile effluents from dyeing represent a major problem, and current adsorption techniques are not environmentally friendly. Natural polysaccharides are highly effective as adsorbents of dye molecules, providing excellent levels of color removal such as guar gum, locust bean gum, and cassia gum [86]. The use of galactomannans for effluent treatment offers significant advantages, such as their natural origin, being renewable, non-toxic, and biodegradable, and having high availability and high adsorption capacity. Furthermore, they do not require any additional chemical or any other treatment other than the use of the adsorbent [228].

### 7.5. Paper Industry

In paper manufacturing, it is important to consider costs, efficiency, environmental issues, and paper strength. The process must be clean due to the high speeds of the machines, with lower grammages and greater use of fillers [229].

Virgin wood pulp fibers have been replaced with secondary fibers in the interest of environmental protection [230]. However, products manufactured with secondary fibers as the main ingredient are weaker due to the drying phases [231]. Paper additives are auxiliary chemical products that have the capacity to increase the strength of paper, making their use necessary when working with secondary fibers [232,233]. In the paper industry, huge amounts of chemicals are consumed during the process of making additives for paper manufacturing, which results in serious environmental pollution. This led to the development of green production methods for paper reinforcing agents [234].

Naturally occurring galactomannans such as locust bean gum and guar gum possess cross-linking properties and good water solubility and can form highly viscous stable aqueous solutions at low concentrations, as well as easily combining with cellulose fibers [235]. Gums have previously been used as additives in papermaking, being added to chemical pulps to accelerate the beating process. Galactomannans improve strength and bond formation by increasing the number of bonds, positively affecting paper strength [229]. The sorption capacity of mannans is related to the similarity they share with the structure of cellulose backbones, so the sorption rate will directly depend on their structure. Independently of the chemical environment, mannans are highly useful in closed papermaking systems and in combination with recycled pulps. For papermaking, galactomannans are used in concentrations ranging from 0.1% to 0.5% of the paper weight. For recycled paper, up to 1% can be used to improve fiber retention and the delamination process [229]. When galactose side groups were removed from the galactomannan structure of guar gum, the sorption rate was significantly improved [235]. That is, natural polysaccharides must be chemically modified in order to be more effective when used as a paper-strengthening agent [236,237]. The strength-enhancing qualities of sheet hemicelluloses can be observed in the recycling of chemical pulps as well. During drying and rewetting processes, the strength of chemical pulps deteriorates as the cell wall structure is modified, leading to less swelling of the fibers. As the swelling is reduced, the flexibility and formability of the fibers is also reduced [238].

Derived mannans are already commercially available and their use also provides a reduction in white water BOD (biological oxygen demand), resistance to sheet detachment, and improved drainage. The use of galactomannans within the paper industry offers multiple positive effects [229].

### 7.6. Other Applications

The application of galactomannans from *G. microphylla* with borax (0.5%) significantly increases water retention and absorption in sandy soil from 15.68 to 38.12%. Galactomannans also help in water treatment, facilitating the aggregation and sedimentation of suspended particles, which favors their elimination [181]. In cherry cultivation, it was observed that when treated with galactomannans 2 weeks before harvest, the fruits effectively reduced the cracking rate. Galactomannans applied as protective films on seeds promote germination with greater efficiency by retaining moisture and nutrients. In addition, gums help transport microorganisms beneficial to plants, improving symbiosis and agricultural growth [239]. Gums have potential for application in the production of silver nanoparticles to detect bioactive compounds in biological systems [240]. In the oil industry, guar gum is used as a filtration-reducing additive in hydraulic fracturing to increase water viscosity and allow the suspension of solid materials. It also facilitates the lubrication and cleaning of wells during drilling [241]. In combination with other compounds, galactomannans are used to manufacture alternative biodegradable materials from renewable sources [242].

## 8. Conclusions

The bioactive properties of galactomannans make them highly valuable compounds with enormous potential for industrial applications, particularly in the formulation of food, pharmaceutical, cosmetic, textile, and paper products. However, their production remains challenging due to the variability in the chemical structure, which depends on both the source and the extraction method used. Various techniques have been explored for their extraction and purification, but many require high energy consumption or involve the use of solvents and large amounts of water. Therefore, it is crucial to develop processes that maximize galactomannan yield while preserving their functional properties and minimizing environmental impact.

The growing demand for natural biopolymers in industry reinforces the importance of continuing to explore the applications of galactomannans. Future research should focus on the design of more efficient and sustainable extraction and purification methods. Additionally, the chemical or enzymatic modification of these polysaccharides could represent new opportunities for creating advanced materials with specific applications in biomedicine and nanotechnology.

As galactomannan production methods improve, their industrial applications are expected to expand further, consolidating their position as a sustainable alternative to synthetic polymers.

## Figures and Tables

**Table 1:** Different sources of galactomannans.

Specie	Origin	Biological Activity	M/G	References
*Ceratonia siliqua* L.	Spain	− Antidiabetic	4:1	[10,11,12,13,14]
*Leucaena Leucocephala*	India	− Pharmaceutical industries	1.3:1	[15]
*Dimorphandra gardneriana*	Brazil	− Drug delivery	-	[16]
*Prosopis affinis*	Uruguay	− Pharmaceutical industries	1:1.5	[6]
*Cyamopsis tetragonolobus* L.	India Italy Brazil Germany	− Stabilizer and emulsifier − Prevents osteoarthritis − Drug delivery − Food industry	2:1	[5,17,18,19,20,21]
*Rhizopogon luteolus*	Turkey	− Anticholinesterase antioxidant	0.81:1.0	[22]
*Caesalpinia spinosa*	Peru China	− Food industry − Pharmaceutical industry	3:1 1.88:1	[12,13,14,23,24,25]
*Ganoderma adspersum*	Turkey	− Food industry	1:1.4	[22]
*Caesalpinia pulcherrima*	Brazil	− Anti-inflammatory	2.18:1	[26]
*Trigonella foenum-graecum L.*	China India	− Antidiabetic − Food industry − Drug delivery − Hypoglycemic	1:1	[27,28,29,30,31,32,33]
*Eremurus hissaricus*	Asia	− Viral diseases	-	[34]
*Bauhinia monandra*	Nigeria	− Food industry	4:1	[35]
*Descurainia Sophia*	Iran	− Pharmaceutical systems	1:1.09	[36]
*Delonix regia*	Nigeria Brazil Mexico	− Pharmaceutical industry − Drug delivery − Anti-inflammatory	4:1	[37,38,39,40]
*Bauhinia vahlii*	India	− Food industry	4.21:1	[41]
*Coffea arabica* L.	India	− Food industry	1:3.5	[42]
*Cassia grandis*	Brazil Cuba	− Matrix of catalytic compounds − Hypoglycemic	-	[43,44,45,46,47]
*Delonix elata*	India	− Food industry	2.55:1	[41]
*Cassia obtusifolia*	China	− Pharmaceutical industry − Drug delivery	1:2.94	[48,49]
*Lallemantia royleana*	Iran	− Hypocholesterolemic	-	[50,51]
*Gleditsia japonica var. delavayi*	China	− Hyperglycemic and Hypolipidemic	2.54:2.66	[7,52,53]
*Cassia tora,*	India	− Drug delivery	5:1	[54]
*Peltophorum pterocarpum*	India	− Food industry	3.03:1	[41]
*Gleditsia caspica*	Iran	− Food industry	1.95:1	[55]
*Adenanthera pavonina* L.	Brazil	− Antidiabetic − Food industry	1.46:1	[56,57,58,59]
*Cassia fistula*	Brazil	− Biomaterial	3.1:1	[60]
*Gleditsia triacanthos* L.	Algeria Argentina	− Food industry	2.86:1	[61,62,63]
*Retama reatam*	Tunez	− Antidiabetic	1.85:1	[64]
*Sesbania cannabina*	China	− Anticancer	2.4:1	[65,66,67,68]
*Trigonella persica*	Iran	− Drug delivery system	5:1	[29]
*Gleditsia sinensis*	China	− Biomaterial − Food industry	3:1 3.55:1	[69,70]
*Sophora japonica f. pendular*	China	− Food industry	4.94:1	[71]
*Coffea canephora*	India	− Food industry	2:1.6	[72]
*Borassus flabellifer*	India	− Biomaterial	1.4:1	[73]
*Gleditsia microphylla*	China	− Food industry	2.77:1	[74]
*Sophora alopecuroides* L.	China	− Pharmaceutical industry	1.48:1	[75]
*Astragalus gombo*	Africa	− Food industry	1.7:1	[76]
*Cassia angustifolia*	India	− Pharmaceutical industry	2.90:1	[77]
*Prosopis ruscifolia*	Argentina	− Pharmaceutical industry	1.6:1	[78]
*Dichrostachys cinerea*	India	− Food industry	1.05:1	[79]

**Table 2:** Different methods of extraction galactomannans.

Extraction Method	Efficiency	Advantages	Disadvantages	References
Hot water	30%	− Most widely used method − Easy to operate − Economic	− Time-consuming − Low recovery rates − Degradation of certain polysaccharides	[107]
Cold plasma	67–122%	− Not require costly reaction chamber − Low energy cost	− Its effect on polysaccharide extraction is still unknown	[30,108]
Three-phase partitioning-based (TPP)	60%	− Easy to operate − Less extraction time − High extraction efficiency	− Use of toxic solvents	[109,110]
Thermal reflux	85–90%	− High extraction efficiency	− Long extraction time − High extraction temperature	[111]
Acid		− High efficiency for hard materials	− Risk of degradation	[112]
Ultrasound-assisted extraction (UAE)	70–90%	− Low energy cost − High extraction efficiency	− Possible molecular damage due to excessive cavitation	[113]
Alkaline		− High extraction efficiency	− Degradation of polysaccharides − Chemical residues	[114]
Microwave-assisted (MAE)	75–85%	− Less extraction time − Less solvent consumption	− Risk of thermal degradation − Not suitable for large-scale production	[115]
Enzyme-assisted	65–85%	− Specific − Less chemical degradation	− Expensive − Requires precise condition control	[114]
Super critical fluids	80–90%	− Green − Without organic waste	− Expensive equipment − High pressure required	[116]
Natural deep eutectic solvents (NADESs)	40%	− High extraction efficiency − Economic	− It is difficult to select the appropriate solvent	[117]

**Table 3:** Different enzymes used for the hydrolysis of galactomannans.

Enzyme	Source	Yield	Substrate	Fermentation	Reference
β-Mannanase	*Penicillium aculeatum* APS1	2807 U/g	Palm kernel cake Soyabean meal	Solid state	[147]
	*Bacillus licheniformis* NK-27	212 U/mL	-	Submerged	[148]
Protease	*Pseudomonas fluorescens* (ATCC 17556)	1.5 U/L	Nutrient broth	Solid state	[149]
			Blood agar	Submerged	
			Skim milk powder	Solid state	
	*Bacillus safensis* CH-25	5.2 U/mL	Casein	Submerged	[150]
Endo-1,4-b-glucanase	*Piptoporus betulinus* CCBAS585	11,300 U/g	Malt extract	Solid state	[151]
Endo-1,4-β-xylanase		1450 U/g			
Endo-1,4-β-mannanase		345 U/g			
1,4-β-Glucosidase		1.4 × 10^6^ U/g			
1,4-β-Xylosidase		106,000 U/g			
1,4-β-Mannosidase		380,000 U/g			
Cellobiohydrolase		88,000 U/g			
Pectinase	*Aspergillus niger* IBT-7	39.1 U/mL	Rice bran	Solid state	[152]
α-Galactosidase	*Debaryomyces hansenii* UFV-1	4.88 U/mL	Lactose	Submerged	[153]

**Table 4:** Different purification techniques of galactomannans.

Technique	Background	Advantages	Disadvantages	Yield (%)	References
Ethanol/ isopropanol precipitation	Removes soluble impurities by precipitating galactomannan	− Simple method − Relatively fast	− Low recovery of polysaccharides − High solvent consumption	70–90%	[117,171,172]
Ion exchange chromatography	Separates biomolecules based on the charge difference between biomolecules and equilibrium ions in the exchanger	− Extensive removal of inorganic ions − High purity of the galactomannans obtained	− Long cycles − High salt consumption − Generates excess wastewater	85–95%	[52,173]
Gel filtration chromatography	Separates polysaccharides based on their molecular weight	− High repeatability − High activity recovery − Low resolution	− Slow process − Use of large amounts of eluent	90–95%	[174,175]
Dialysis	Removes low molecular weight contaminants	− Excellent for obtaining high purity products − Does not require organic solvents	− Slow process. − Specialized equipment and scale limitations.	80–90%	[6]
Ultrafiltration	Concentrates galactomannan and removes small, unwanted molecules.	− Solvent-free method − Efficient for removing small contaminants	− Requires high pressure − High cost of equipment and membrane maintenance	70–90%	[176]

**Table 5:** Different techniques for characterization of galactomannans.

Technique	Findings	References
Fourier transform infrared spectroscopy (FTIR)	Galactomannan obtained from *Adenanthera pavonina* L. retains its characteristic monosaccharides after purification, such as mannose and galactose. The presence of α-D-galactopyranose and β-D-mannopyranose units was observed	[58,59]
Nuclear magnetic resonance (^1^H-NMR and ^13^C-NMR)	Galactomannan extracted from *Prosopis affinis* shows a M/G ratio of ~1.5 with a main structure of β-(1,4) mannan backbone adorned with galactosyl residues attached through α-(1,6) linkages	[6]
Gas chromatography (GC)	Reports different monosaccharide compositions for *Angelica sinensis*, most of these polysaccharides including mannose, rhamnose, arabinose, glucose, galactose, and galacturonic acid with various proportions	[186]
High-performance gel filtration chromatography (HPGFC)	Glucose is the main monosaccharide unit of *Lepidium meyenii* extracts, composed of rhamnose, arabinose, glucose, and galactose	[187]
Scanning electron microscopy (SEM)	Galactomannans extracted from *Trigonellafoenum-graecum L.* show structural integrity and smooth surfaces, being candidates for film fabrication and drug delivery	[3]
X-Ray diffraction (XRD)	Galactomannan extracted from *Adenanthera pavonina* L. has a semi-crystalline structure after precipitation with ethyl alcohol	[58]
Thermogravimetry (TGA)	*Trigonellafoenum-graecum* extracts show a high number of carboxyl groups	[3]
Differential scanning calorimetry (DSC)	*Trigonellafoenum-graecum* extracts are stable at high temperatures and show the ability to retain moisture for long periods	[3]
Rheometry and viscosity	At higher temperatures, the polymer chains of *S. japonica* degrade, reducing the intrinsic viscosity and the average molecular mass of the viscosity	[117]
Dynamic light scattering (DLS)	The value of the structure-sensitive parameter was 1.33, suggesting that *Cassia obtusifolia* extracts adopted a random coil conformation in solution	[48]
High-performance thin-layer chromatography (HPTLC)	*Senna tora* hydrolysate showed spots identified as galactose and mannose and an average M/G ratio of 5.1 was observed	[188]

## Data Availability

No new data were created or analyzed in this study. Data sharing is not applicable to this article.
